# Illusory ownership of an invisible body reduces autonomic and subjective social anxiety responses

**DOI:** 10.1038/srep09831

**Published:** 2015-04-23

**Authors:** Arvid Guterstam, Zakaryah Abdulkarim, H. Henrik Ehrsson

**Affiliations:** 1 Department of Neuroscience, Karolinska Institutet, Retzius väg 8, SE-171 77 Stockholm, Sweden

## Abstract

What is it like to be invisible? This question has long fascinated man and has been the central theme of many classic literary works. Recent advances in materials science suggest that invisibility cloaking of the human body may be possible in the not-so-distant future. However, it remains unknown how invisibility affects body perception and embodied cognition. To address these questions, we developed a perceptual illusion of having an entire invisible body. Through a series of experiments, we characterized the multisensory rules that govern the elicitation of the illusion and show that the experience of having an invisible body reduces the social anxiety response to standing in front of an audience. This study provides an experimental model of what it is like to be invisible and shows that this experience affects bodily self-perception and social cognition.

The power of becoming invisible has captured the imagination of writers and philosophers for millennia^1^,^2^,^3^,^4^. In H.G. Wells’s science fiction novel *The Invisible Man* (1897), the protagonist invents a method to change a body’s refractory index to that of air, rendering it invisible, and then he successfully carries out the procedure on himself (but he unfortunately fails to reverse it). Intriguingly, advances in materials science have demonstrated that invisibility cloaking of large-scale objects, not too dissimilar from what Wells envisioned, is becoming reality^5^,^6^. In fact, physicists recently reported the first successful cloaking of living animals by demonstrating the effect on a cat and a fish^7^. The prospect of using these techniques on humans raises important neuroscientific questions: How does invisibility affect self-perception of one’s body? Are there any cognitive “side effects” of having an invisibly body? In this study, we present an experimental model for investigating these issues. We first show that it is possible to elicit the perceptual illusion of owning an invisible body, and then use this illusion to examine if the feeling of invisibility affects aspects of social and affective cognition.

Perceptual body illusions have, over the past decade, become important tools for investigating the neural mechanisms that underlie the feeling of body ownership^8^,^9^,^10^,^11^. The seminal report of the rubber hand illusion demonstrated that the sight of a rubber hand being touched in synchrony with touches applied to one’s hidden real hand elicits an illusion that the rubber hand is part of one’s own body^8^. The illusory self-attribution of the rubber hand is dependent on the integration of temporally and spatially congruent visual, tactile, and proprioceptive signals^8^,^10^,^12^ in multisensory cortical areas in the premotor, intraparietal, and cerebellar cortices^9^,^13^,^14^. It has been assumed that the visual presence of a rubber hand is important for the ownership illusion to arise^10^,^15^; however, in contrast to this notion, we recently showed that the illusion can in fact be induced for an invisible hand^16^. The invisible hand illusion was elicited through the use of synchronized touches delivered to the hidden real hand and to a hand-shaped volume of empty space in front of the participant. This setup resulted in the referral of tactile sensations to the empty space and the perception of having an invisible hand, which was associated with increased neural activity in the multisensory regions that were found to be active during the rubber hand illusion^16^. This observation provokes an intriguing question: Can the illusion of having an invisible limb be extended to an entire invisible body?

In support of this hypothesis, previous studies have shown that the illusion of owning a rubber hand can be extended to an entire body^17^. In the full-body illusion, the participants view a mannequin’s body being touched through a set of head-mounted displays (HMDs) while they receive correlated tactile stimulation on their real body (Figure 1, right panel). This setup results in the illusory experience of the mannequin’s body being one’s own body^17^,^18^, which is reflected by increased neural activity in the set of areas that are implicated in the rubber hand and invisible hand illusions^19^. Based on these findings, we hypothesized that the multisensory integrative mechanisms that underlie the invisible hand illusion can be generalized to the illusion of having an entire invisible body. To test this prediction, we modified the experimental setup of the mannequin illusion by removing the mannequin’s body and applying the touches to a discrete volume of empty space that represented an invisible body (Figure 1, left panel). We used a combination of subjective questionnaires^8^ and physiological measures of one’s response to threatening stimuli to quantify the illusion^14^. In Experiments 1a and 1b, we found that the “invisible body illusion” can indeed be elicited and that it obeys specific temporal and spatial multisensory congruence principles. Experiments 2a and 2b directly compared this illusion with that of owning a mannequin’s body and showed that the two illusions are correlated and equally strong. In Experiment 3, we used a body-image task to show that experiencing the invisible body illusion involves perceiving one’s body image as more transparent, in contrast to the mannequin illusion. In Experiment 4, we tested the hypothesis that the feeling of invisibility affects social cognition. To this end, we measured heart rate and subjectively perceived stress in response to a socially stressful stimuli (standing in front of an audience) and found that owning an invisible body reduces the social anxiety associated with this situation. In conclusion, we characterize the first full-body illusion that involves a non-physical body and use this illusion as an experimental model for investigating the cognitive effects of invisibly cloaking of humans.

## Methods

### Participants

One hundred twenty-five naïve, healthy volunteers participated in the study. None of the subjects took part in more than one experiment. Based on the results of previous studies using similar measurements^16^,^20^,^21^, we estimated that the sample size necessary to achieve statistical significance would be approximately 20 participants. We recruited 25 participants in each experiment because, based on previous experience, we estimated that about 20% of the scheduled subjects would not show up. In Experiment 4, we recruited six additional participants because of a technical issue with the heart rate data acquisition (see below). The data collection was stopped when the originally scheduled participants had been tested (no-shows were excluded from any further analysis). All of the participants were instructed to wear a pair of trousers and a t-shirt. The study was approved by the Ethical Review board at Karolinska Institutet, the methods were carried out in accordance with the approved guidelines, and all participants gave their written informed consent.

### Experimental setup and illusion induction procedure

**Figure 1:**
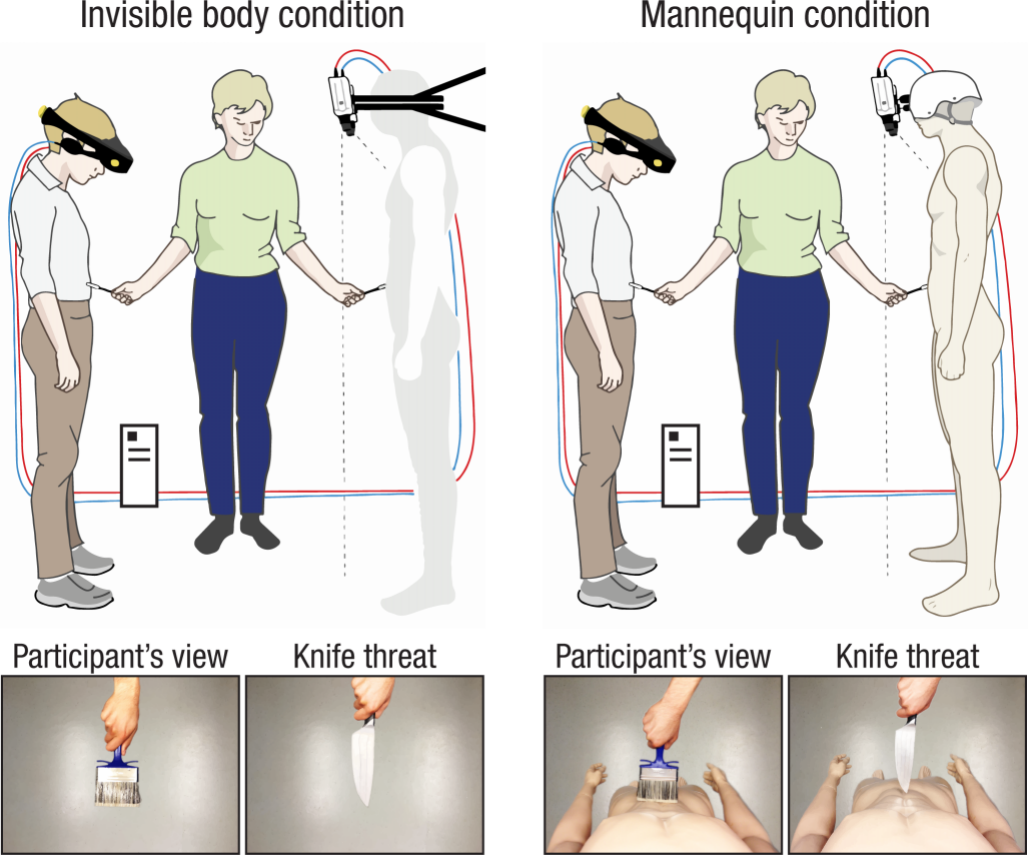
The experimental setup of the invisible body condition (left panel) and the mannequin condition (right panel). The participants were fitted with a set of head-mounted displays (HMDs) that showed the real-time 3-D video feed of a pair of downward-facing cameras that were mounted on the wall (left panel) or on the head of a mannequin (right panel). The experimenter applied touches to the participants’ body and the corresponding body part of the invisible body/mannequin using a paintbrush. The grayed-out body in the left panel illustrates the discrete portion of empty space that was meant to represent the invisible body. Two sample frames of the actual visual stimuli presented in HMDs are shown below, featuring the brushing procedure and knife threat event for the two conditions, respectively.

The participants were asked to stand in an upright position with their head tilted and to look down at their body. They were then fitted with a set of HMDs (VR1280, Virtual Research Systems Inc., California, USA; 1280 x 1024 display resolution per eye, 60° field of view). The HMDs presented the participants with video streams of two high-performance, industrial, digital cameras (Stingray F-046, Allied Vision Technologies, Stadtroda, Germany; 780 x 580 resolution, 60 fps) that were attached side by side with a custom-made tripod mounted on the wall at the level of the participant’s head and that faced the floor (Figure 1, left panel). The video streams from the left and right cameras were transmitted to the left and right displays of the HMDs, respectively, through a computer that ran in-house, 3-D, video-displaying software. The total video delay was non-noticeable (89 ms). Thus, through the HMDs, the participants viewed the empty space below the cameras in 3-D and in approximately real-time. To induce the illusion, the experimenter stroked the participant’s body with a large paintbrush while simultaneously moving another paintbrush in the corresponding location in the empty space below the cameras, as if he were touching an “invisible body” that was in this location (illustrated by the grey body in Figure 1, left panel). The experimenter applied touches to five different body parts: the abdomen, the left and right lower arms, and the left and right lower legs and feet. The duration of each individual brushstroke was 1 s and the time between the offset of one touch and the onset of the next touch was 1.5 s (to ensure that there were no overlaps between the seen and felt touches in the asynchronous condition). The brushstrokes were delivered in the following pre-determined sequence (A = abdomen, RLA = right lower arm, LLA = left lower arm, RLF = right lower leg and foot, LLF = left lower leg and foot):
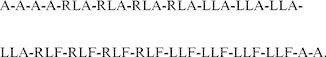


To identify the portions of empty space representing the abdomen, arms, legs and feet of the invisible body, we used the mannequin as a template. Visual landmarks were placed outside the field of view of the cameras (i.e., hidden from the participants’ view) that indicated the starting and stopping points of the brushstrokes for each individual body part. We then removed the mannequin and the experimenter (ZA) practiced extensively on delivering the brushstrokes in the empty space, until he could deliver spatially well-defined strokes with a high level of synchrony and stability. In the questionnaire experiments (Experiments 1a, 2a, and 3), the duration of one experimental block was 2 min, and each condition was repeated only once^16^,^20^,^21^. In the SCR experiments (Experiments 1b and 2b), the duration of one block was 1 min, and each condition was repeated three times in a pseudo-randomized order^16^,^20^,^21^. In Experiment 4, which included measures of both heart rate and questionnaire responses, the block duration was 1 min, and each condition was repeated twice. The heart rate was recorded in the first set of repetitions, and the questionnaire responses were given in the second set of repetitions. The presentation order of the experimental conditions was balanced across participants in all of the experiments.

### Experiments 1a and 1b: Temporal and spatial congruence

The aim of Experiments 1a and 1b was to provide subjective and objective evidence for the invisible body illusion. In our previous study, we demonstrated that the illusion of having a single invisible limb is dependent on spatio-temporal congruence of visual and tactile signals^16^. We therefore hypothesized that the brushstrokes applied to the empty space and the touches delivered to the participant’s body would need to be synchronous and spatially aligned for the invisible body illusion to be elicited. In two independent experiments, we combined questionnaire (Experiment 1a) with SCR measurements (Experiment 1b) and included the following three experimental conditions: synchronous (illusion condition), asynchronous (control), and spatially incongruent (control) visual and tactile stimulation. In the asynchronous condition, the brushstrokes observed in the empty space were delayed by 1.25 s with respect to the tactile stimulation, while keeping all the other factors constant. The incongruent condition consisted of synchronous visuo-tactile stimulation; however, the touches on the participant’s real body were applied in the opposite direction and on another body part. The questionnaire data were first analyzed using a 3 × 2 ANOVA with the factors condition (synchronous, asynchronous, incongruent) and statement type (illusion, control) to examine the main effect of condition. To investigate the contrasts synchronous vs. asynchronous and synchronous vs. incongruent, we ran two separate 2 × 2 ANOVAs for the factors condition (synchronous, asynchronous or synchronous, incongruent) and statement type (illusion, control). The SCR data were analyzed with a one-way ANOVA and two paired t-tests for the planned comparisons of synchronous vs. asynchronous and synchronous vs. incongruent. We included 15 participants in Experiment 1a (six females, mean age 27 ± 7 years) and 22 participants in Experiment 1b (19 females, mean age 22 ± 4 years).

### Experiments 2a and 2b: Invisible versus solid body

**Figure 2:**
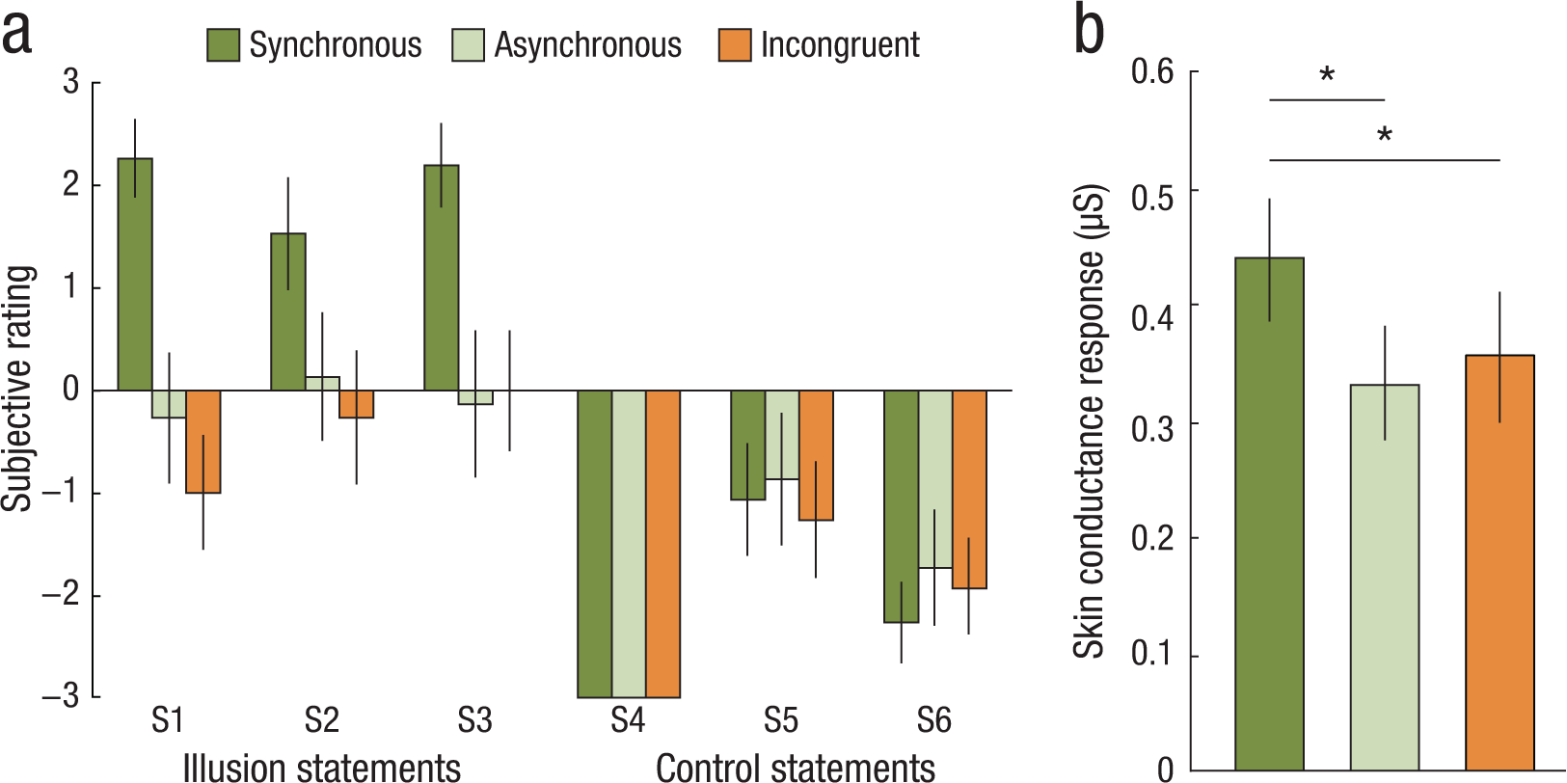
Results of Experiments 1a and 1b. (**a**) The results of Experiment 1a showed that the participants rated the statements reflecting the illusory experience (S1-S3) significantly higher in the synchronous illusion condition than in the asynchronous and spatially incongruent control conditions. No such differences were observed for the control statements (S4-S6). (**b**) The results of Experiment 1b showed that the skin conductance response (SCR) evoked by a knife entering the field of view and threatening the invisible body was significantly stronger in the synchronous condition than in the asynchronous and spatially incongruent conditions. Together, these results suggest that the invisible body illusion is dependent on temporally and spatially congruent visuo-tactile stimulation. *P < 0.05.

**Figure 3:**
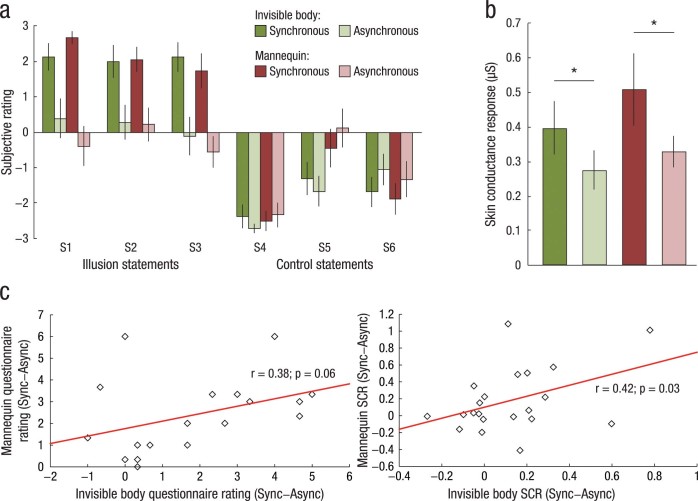
Results of Experiments 2a and 2b. (**a**) The results of Experiment 2a show that strength of the invisible body illusion was equal to that of the mannequin illusion (the three-way interaction of body type × visuo-tactile temporal congruence × statement type was non-significant). (**b**) The SCR results of Experiment 2b show a significant main effect of visuo-tactile temporal congruence and a non-significant interaction of body type × visuo-tactile temporal congruence, corroborating the conclusion that the invisible body and mannequin illusions are of similar strengths. (**c**) There was a positive correlation between the magnitudes of the invisible body and mannequin illusions in terms of subjective ratings (left graph) and threat-evoked SCRs (right graph). These results imply that the elicitation of the illusions is dependent on analogous neural processes. *P < 0.05.

The aim of Experiments 2a and 2b was to compare the invisible body illusion with the previously published “body-swap” mannequin illusion, in which the participants experience ownership of a mannequin’s body^17^,^19^. The results of Experiments 1a and 1b demonstrated that the invisible body illusion appears to obey the same spatio-temporal multisensory rules as the mannequin illusion (Figure 2). We therefore predicted that the subjective strength and the threat-evoked SCR of these two full-body illusions would be correlated and of similar magnitude. In two independent experiments, we compared the illusion strengths in terms of questionnaire ratings (Experiment 2a) and threat-evoked SCR (Experiment 2b) using a 2 × 2 factorial design, with the main factors being visuo-tactile temporal congruence (synchronous, asynchronous) and body type (invisible, mannequin). In the two mannequin conditions, the body of a male mannequin was placed below the cameras, keeping all of the other factors equivalent to the invisible body conditions (Figure 1, right panel). The questionnaire data were analyzed using a 2 × 2 × 2 ANOVA, with the factors being body type (invisible, mannequin), visuo-tactile temporal congruence (synchronous, asynchronous) and statement type (illusion, control). The SCR data were entered into a 2 × 2 ANOVA, with the factors being body type (invisible, mannequin) and visuo-tactile temporal congruence (synchronous, asynchronous) (Figure 3). Eighteen subjects participated in Experiment 2a (13 females, mean age 25 ± 10 years), and 21 participated in Experiment 2b (7 females, mean age 27 ± 10 years).

### Experiment 3: Effects on the perceived body image

**Figure 4:**
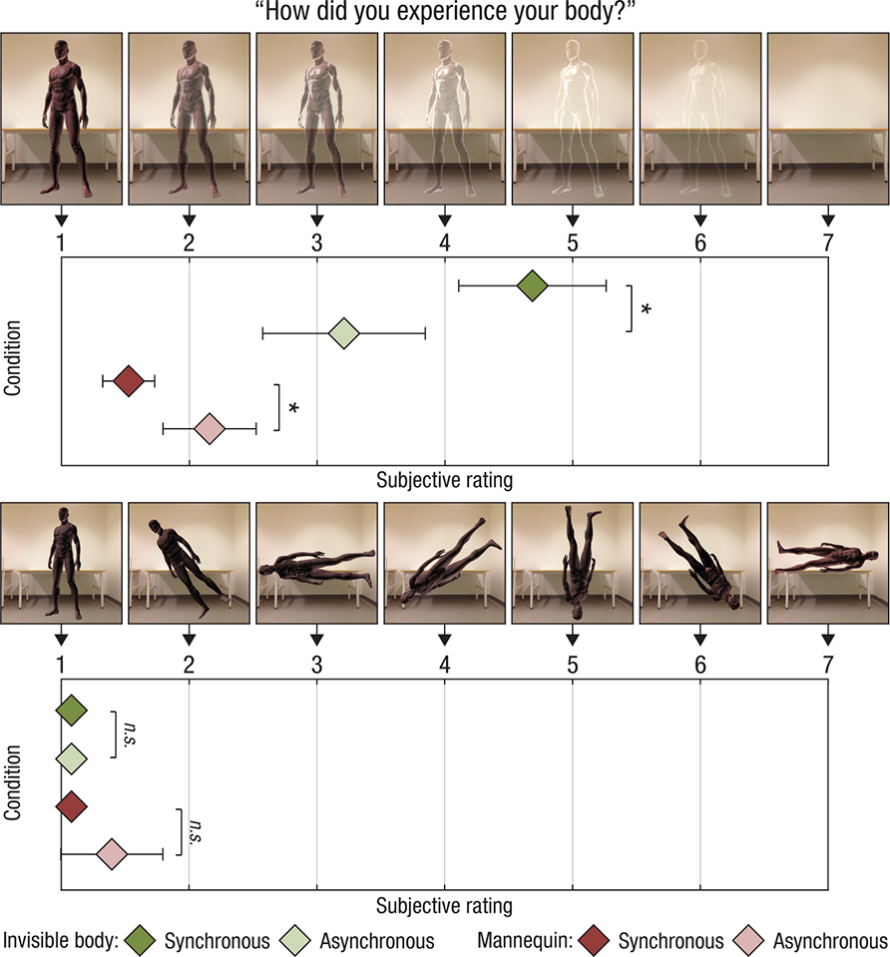
Results of Experiment 3. The upper panel shows that the participants reported that the invisible body illusion induced a significant shift in the perceived body image toward greater transparency, in contrast to the mannequin illusion’s effect (there was a significant body type × visuo-tactile temporal congruence interaction). The lower panel displays the results of the control task, in which the participants estimated the perceived orientation of their body, which revealed no significant differences between conditions. *P < 0.05.

The aim of Experiment 3 was to quantify potential effects on the conscious body image, which was not fully captured by the questionnaire and SCR measurements. To address this question, we developed a body image test consisting of a perceptual matching task that assessed the participants' perceived degree of body transparency (see *Body image task* below and Figure 4, upper panel). We used a 2 × 2 factorial design, with the main factors being body type (invisible, mannequin) and visuo-tactile temporal congruence (synchronous, asynchronous). The inclusion of the mannequin conditions allowed us to control for unspecific effects related to experiencing a full-body illusion. We hypothesized that we would find a significantly higher rating in the synchronous condition than in the asynchronous condition for the invisible body, but we did not expect to find such a difference in the mannequin conditions (i.e., a significant body type × visuo-tactile temporal congruence interaction). Additionally, the participants performed a separate perceptual matching task to control for their suggestibility and task compliance. In this control task, the spatial orientation of the body relative to the environment was systematically altered (Figure 4, upper panel). We did not expect significant differences between any of the conditions in this control task. We included 20 participants in Experiment 3 (10 females, mean age 28 ± 7 years).

### Experiment 4: Effects on social anxiety

Studies on social anxiety within virtual environments have shown that exposing an individual to virtual social situations elicits anxiety responses that mimic the responses to analogue real-world social situations^22^,^23^,^24^. Standing in front of an audience is generally acknowledged as a stressful event and is associated with increased heart rate and levels of social anxiety^25^,^26^. The aim of the final experiment was to test the hypothesis that the feeling of invisibility would reduce the perceived anxiety related to experiencing a stressful social situation. We based this prediction on the assumption that if the body is represented as an invisible entity, it will be represented as being invisible to outside observers as well, which, in turn, should reduce the brain’s social anxiety response to being the center of other people’s attention. To test this hypothesis, we recorded the participants’ heart rate and their subjectively rated stress level in response to standing in front of an audience after exposure to 1 min of visuo-tactile stimulation. Again, we employed a 2 × 2 factorial design, with the main factors being body type (invisible, mannequin) and visuo-tactile temporal congruence (synchronous, asynchronous). First, we repeated the conditions once in a randomized order while recording the heart rate. We then repeated each condition again and asked the participants to verbally report their level of stress when looking up and seeing the crowd, using a visual analogue scale (presented on the HMDs immediately after each repetition) ranging from 0 (“*I felt fully relaxed.*”) to 100 (“*I felt extremely stressed.*”). We hypothesized that the perceived level of stress would only be elevated in the condition in which the participants experienced being physically present in front of the crowd (i.e., during the mannequin synchronous condition). We thus predicted a significant visuo-tactile temporal congruence × body type interaction to be driven by a significant difference between invisible body synchronous vs. mannequin synchronous, whereas we expected the contrast invisible body asynchronous vs. mannequin asynchronous to be non-significant*.* We included 29 participants (10 females, mean age 28 ± 7 years) in the experiment. Six subjects had to be excluded from the heart rate data analysis owing to severe artifacts in the ECG recording.

### Questionnaires

We used questionnaires to quantify the subjective experience associated with the illusion in Experiments 1a and 2a^8^. Immediately following each experimental condition, the participants were asked to remove the HMDs and rate six different statements (Table 1) concerning their experience using a seven-point Likert scale, ranging from -3 (“*I completely disagree*”) to +3 (“*I completely agree*”), with 0 corresponding to “*I neither agree nor disagree.*” Three of the statements (S1-S3) examined the perception of the illusion, and the other three statements (S4-S6) were designed to control for suggestibility and task compliance. The statistical analyses of the questionnaire data were performed on the average rating of the illusion statements (S1-S3) and control statements (S4-S6).

### Skin conductance responses and knife threat

We threatened the invisible body with a knife and measured the evoked skin conductance responses (SCRs) in Experiments 1b and 2b to provide an objective physiological measure of the illusion. Previous studies have shown that the threat-evoked SCR is a reliable proxy for changes in bodily self-attribution^16^,^17^,^20^,^21^,^27^ and that a stronger feeling of ownership of a seen limb is directly related to increased threat-evoked neuronal responses in the areas that reflect pain anticipation^14^,^28^. Here, a hand that was holding a knife entered the participant’s field of view from above and performed a slow, continuous motion toward the abdomen of the invisible body (or the mannequin’s body in Experiment 2b), as illustrated in (Figure 1). The knife stopped just before “hitting” the abdomen of the invisible/mannequin body, changed direction (-180°) and then disappeared out of the field of view in the same motion that it entered. The duration of the entire event was approximately 2 s. The threat-evoked SCR was identified as the peak of conductance that occurred within 5 s of the onset of the threat stimulus (from the first moment that the knife entered the participant’s visual field) and was flagged in the SCR recording file. The investigator who performed the analysis was blind to the condition (i.e., illusion or control). We used a Biopac System MP150 (Goleta, California, USA) to record the SCR, and all of the data acquisition parameters and recording procedures were identical to those used in our previous studies^16^,^17^,^20^,^21^,^29^.

### Body image task

For Experiment 3, we developed a test to examine the potential effects of the illusion on perceived body image. The test consisted of a perceptual matching task in which the participants were presented with a range of schematic drawings of seven bodies with different degrees of invisibility (Figure 4, upper panel). The series of bodies constituted a seven-point scale, ranging from a solid body (1), to increasingly transparent bodies (2–4), to a hollow body that merely had contours (5–6), to a completely invisible body (7). The participants’ instructions were the following: “*How did you experience your body? Below you will find seven schematic drawings of your body during the experiment. Please select the body below which best corresponds to your experience*.” To control for suggestibility and task compliance, the participants performed a separate perceptual matching task in which the spatial orientation of the body relative to the environment, rather than the transparency of the body, was systematically altered. In this task, the seven-point scale represented different angular rotations of a solid body (Figure 4, lower panel).

### Heart rate measure and social stress stimuli

**Figure 5:**
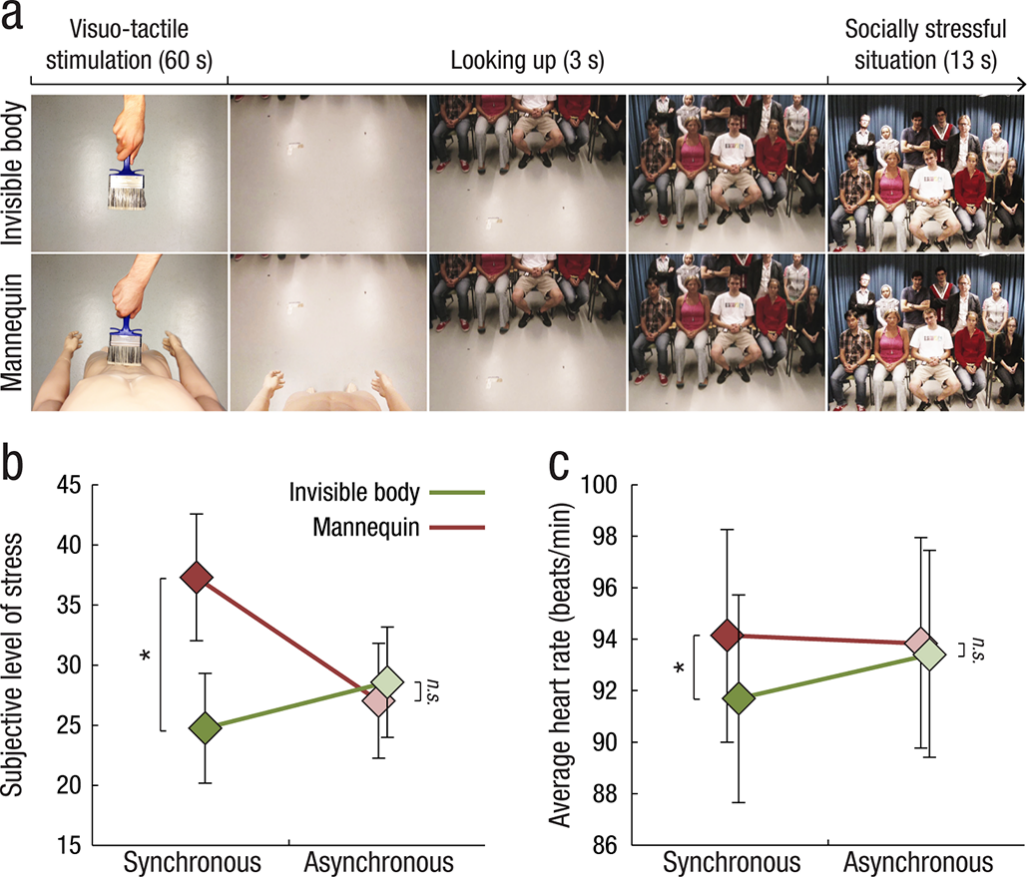
Setup and results of Experiment 4. (**a**) The experiment timings and five representative frames from the visual stimuli are shown. Following 60 s of synchronous or asynchronous visuo-tactile stimulation that featured the invisible body or the mannequin’s body, the participants slowly lifted their gaze to discover that they were standing in front of an audience. The audience consisted of 11 scientists who were instructed to look directly at the participant (i.e., the cameras providing visual input to the HMDs) with a stern, serious face. The participants’ heart rate and subjective level of stress were measured. (**b**) The invisible body illusion was associated with significantly lower stress ratings than were found in the mannequin illusion. No significant difference was observed in the asynchronous control conditions. (**c**) In accordance with the subjective data, the heart rate response was significantly lower in the invisible body condition than in the mannequin condition - but only in the synchronous illusion condition. These results suggest that the illusion of owning an invisible body reduces the social anxiety associated with the experience of standing in front of an audience.

In Experiment 4, we recorded the participants’ heart rate while they were exposed to a stressful social situation to examine the potential effects of the illusion on social anxiety. The “social stress event” consisted of standing in front of a crowd of unknown people for 13 s, which immediately followed 1 min of visuo-tactile stimulation (see Figure 5a). The crowd comprised of 11 lab group members who were instructed to put on a skeptical and serious-looking face and look directly toward the position of the cameras (i.e., the position from which the participants viewed the room). The specific time interval of 13 s was the result of approximations and informal pilot experiments: the primary aim was to maintain the duration of the social stress event sufficiently long for the participants to comprehend the situation and have time to inspect the faces of the crowd of strangers, but at the same time not too long, because one would expect that the illusory feeling of invisibility and its potential stress-reducing effect decreases over time when not maintained through visuo-tactile stimulation. For practical reasons, we used pre-recorded 3-D videos of the visual stimuli (using two identical cameras mounted in parallel 8 cm apart; CamOne Infinity HD, resolution 1920 × 1080, Touratech AG, Germany) instead of the real-time setup used in Experiments 1–3. The experimenter wore headphones and listened to a pre-recorded audio track to synchronize the tactile stimulation with the videos. The participants were blind-folded and wore ear plugs when they entered the experiment room. This precaution was taken in order to minimize the risk of the participants discovering that experiment room was in fact empty of a crowd of people and that the visual stimuli were pre-recorded. In order to synchronize the lifting of the participants’ gaze with the prerecorded videos, the participants were instructed that at certain occasions during the experiment the experimenter would gently hold and lift the HMDs and that they should follow this motion with their head. Thus, by lightly holding and lifting the HMDs in a single controlled movement, the experimenter slowly lifted the participant’s gaze in synchrony with the pre-recorded visual input. Unfortunately, we did not formally quantify the feeling of presence in the virtual environment, as one anonymous reviewer pointed out. Anecdotally, however, most participants were surprised to find the experiment room empty of people when removing the HMDs after concluding the experiment, and the results of the experiment speak in favor of a high degree of presence felt (see Results). Nevertheless, we recommend that future studies include a post-experiment questionnaire regarding the participants’ feeling of presence in the spatial environment presented in the HMDs.

We used the Biopac System MP150 (Goleta, USA) and three electrodes (attached the left arm, right arm, and left foot of the participant) to register a single-lead electrocardiogram (ECG). The R-wave detector function, which removes any components of the waveform that might be mistaken for peaks, was activated to optimize the ECG data for heart-rate calculation. The heart rate was calculated for every heartbeat based on the R-R interval with respect to the preceding beat. As a measure of general autonomic arousal in response to the social stress event, we calculated the mean heart rate for the 13 s that corresponded to the social stress event. To control for potential condition-specific effects on the heart rate that are unrelated to standing in front of the crowd, we analyzed the mean heart rate for the 13 s *preceding* the social stress event onset.

### Statistical analysis

The Kolmogorov–Smirnoff test was used to check the normality of the data. For normally distributed data sets, we used *t*-tests and repeated-measures ANOVAs. For data sets that were not normally distributed, we used the non-parametric Wilcoxon signed-rank test. Although the data were not normally distributed, we investigated the interaction effects between two main factors in a 2 × 2 factorial design and calculated the “nonparametric interaction” (referred to as “interaction”) by calculating the numeric difference between the two levels of each factor and then statistically comparing these differences using a two-tailed Wilcoxon signed-rank test. We used two-tailed tests for all the analyses except the ones for which we had strong *a priori* hypotheses (the planned pair-wise comparisons in Experiment 1b and correlation analyses in Experiments 2a and 2b), in which one-tailed tests were used. The alpha was always set at 5%.

## Results

### Experiments 1a and 1b: Temporal and spatial congruence necessary for the illusion

In Experiments 1a and 1b, we compared synchronous, asynchronous and spatially incongruent brushstrokes on the participant’s body and on the portion of empty space that represented the invisible body. The main effects of condition (F_2,28 _ = 8.12, *P* < 0.01) and statement type (F_1,14 _ = 52.33, *P* < 0.01), as well as their interaction (F_2,28 _ = 13.21, *P* < 0.01), were all significant in a 3 × 2 ANOVA. Specifically, our results showed that participants in the synchronous condition affirmed the statements in the questionnaires that reflected the illusory percept more strongly than they affirmed the control statements, as compared to the asynchronous (significant interaction condition × statement type: F_1,14_ = 27.53, *P* < 0.01) and incongruent (significant interaction condition × statement type: F_1,14_ = 17.12, *P* < 0.01) conditions. Moreover, the threat-evoked SCR was significantly higher in the synchronous condition than in the asynchronous and incongruent conditions (F_2,42_ = 3.44, *P* = 0.04, one-way ANOVA; synchronous vs. asynchronous, *t* = 2.39, *P* = 0.01; synchronous vs. incongruent, *t* = 1.74, *P* = 0.05, planned, one-tailed *t*-tests). Thus, temporal and spatial congruence of the visual and tactile signals are necessary for the elicitation of the illusion.

### Experiments 2a and 2b: Invisible versus solid body

In Experiments 2a and 2b, we directly compared the invisible body illusion and the mannequin illusion in terms of subjective ratings and threat-evoked SCR, using a 2 × 2 factorial design, with the factors being body type (invisible, mannequin) and visuo-tactile temporal congruence (synchronous, asynchronous). The results of the questionnaire Experiment 2a showed that the effect of body type (F_1,17_ = 0.80, *P* = 0.39) and the three-way interaction of body type × visuo-tactile temporal congruence × statement type (F_1,17_ = 2.64, *P* = 0.12) were non-significant, which was in accordance with our *a priori* hypothesis that both illusions would engage similar multisensory processes (Figure 3a). This interpretation was further strengthened by the observed correlation between the average illusion statement score for the invisible body and for the mannequin (using the synchronous vs. asynchronous difference for the respective illusions as variables) (Figure 3c: *r* = 0.38; *P* = 0.06, one-tailed Pearson correlation). The effect of visuo-tactile temporal congruence was significant (F_1,17_ = 17.27, *P* < 0.01), which replicated the results of Experiment 1a. In the SCR Experiment 2b, we found a significant effect of body type (F_1,20_ = 7.69, *P* = 0.01) and visuo-tactile temporal congruence (F_1,20_ = 6.89, *P* = 0.02); however, their interaction of body type × visuo-tactile temporal congruence was non-significant (F_1,20_ = 0.56, *P* = 0.46) (Figure 3b). A *post hoc* *t*-test revealed a significantly greater SCR in the synchronous mannequin compared to the synchronous invisible body condition (*t* = −2.07, *P* = 0.05, two-tailed paired *t*-test). The correlation between the threat-evoked SCR in the invisible body condition and the mannequin condition (again, using the synchronous vs. asynchronous difference as variables) was significant (Figure 3c: *r* = 0.42, *P* = 0.03, one-tailed Pearson correlation), which is compatible with the questionnaire data correlation that showed a trend toward significance. Together, Experiments 2a and 2b provide behavioral evidence that the invisible body illusion and the mannequin illusion recruit similar central multisensory processes^17^,^19^.

### Experiment 3: The feeling of invisibility affects the perceived body image

The aim of Experiment 3 was to characterize the effect of the illusion on perceived body image. To this end, we developed a task in which the participants were shown bodies of different degrees of invisibility and asked to select the one that best corresponded to their subjective experience (Figure 4, upper panel). As hypothesized, the results revealed a significant interaction of body type × visuo-tactile temporal congruence (*Z* = −3.16, *P* < 0.01). The synchronous condition generated significantly higher ratings than the asynchronous condition for the invisible body (4.55 vs. 3.30; *Z* = −2.18, *P* = 0.03, two-tailed Wilcoxon signed-rank test). Unexpectedly, we observed a significant synchronous vs. asynchronous difference in the mannequin condition, but this difference was in the opposite direction (1.50 versus 2.25; *Z* = −2.60, *P* < 0.01, two-tailed Wilcoxon signed-rank test). The results of the control task, designed to control for general suggestibility and task compliance, revealed no significant interaction of body type × visuo-tactile temporal congruence (*Z* = −0.82 p = 0.41; Figure 4, lower panel). Together, these results suggest that the invisible body illusion involves a significant change in the perceived physical quality of the body by inducing a shift in perception from the natural solid body image toward a transparent and hollow one.

### Experiment 4: Feeling invisible reduces social anxiety responses

Finally, in Experiment 4, we quantified the subjectively perceived stress level and measured heart rate in response to a socially stressful situation (Figure 5a). We hypothesized that the experience of invisibility would affect not only the perception of one’s own body but also the manner in which the brain processes the attention of others toward the self. Thus, if the participants truly experience invisibility, their body should be represented as invisible to others individuals as well, which presumably reduces anxiety responses related to being in the “center of attention.” To test this hypothesis, we placed the participants in front of an audience of serious-looking strangers, following a 1-min period of visuo-tactile stimulation. As predicted, the reported level of stress was significantly lower in the invisible body synchronous condition than in the mannequin synchronous condition (*t* = −2.54; *P* = 0.02, two-tailed *t*-test; Figure 5b). Importantly, there was no significant difference between the asynchronous conditions (*t* = 0.40; *P* = 0.69, two-tailed *t*-test), and there was a marginally significant interaction of visuo-tactile temporal congruence × body type (F_1,28_ = 4.17; *P* = 0.05). In line with the subjective reports, we observed significantly lower heart rates in the invisible body synchronous condition than in the mannequin synchronous condition (*t* = −2.37; *P* = 0.03, two-tailed *t*-test; Figure 5c), but there was no significant difference between the asynchronous conditions (*t* = −0.35; *P* = 0.73, two-tailed *t*-test) (although the interaction of visuo-tactile temporal congruence × body type did not reach significance, F_1,22_ = 2.60; *P* = 0.16). There were no significant differences in heart rate during the 13 s preceding the onset of the stressful social event (invisible synchronous vs. mannequin synchronous, *P = * 0.98; invisible asynchronous vs. mannequin asynchronous, *P = * 0.25, two-tailed *t*-tests), suggesting that there were no general effects of condition on the heart rate that could confound the results.

## Discussion

Our results demonstrate that healthy individuals can experience the illusion of owning an invisible full-body. This perceptual illusion arises when participants observe (through a set of HMDs) a paintbrush moving in an empty space and defining the contours of an invisible body, while receiving simultaneous touches on the corresponding parts of their real body that is hidden from view. Two main conclusions can be drawn from the data. First, the invisible body illusion depends on the integration of visual and tactile signals according to basic multisensory integration principles of temporal and spatial congruence (Experiments 1a and 1b), similar to the mechanisms involved in generating illusory ownership of a mannequin’s body (Experiments 2a and 2b). Second, experiencing the invisible body illusion has unique effects on body perception and social-affective cognition, respectively, such as it inducing a shift in the perceived body image toward near-complete transparency (Experiment 3) and reducing social anxiety when standing in front of an audience (Experiment 4). Collectively, these findings extend our understanding of the central processes that underlie body ownership and its interactions with social and affective processes and offer potentially novel treatment strategies for social anxiety disorder.

The existence of the invisible body illusion has implications for our understanding of the multisensory mechanisms involved in the invisible hand illusion^16^ and the mannequin illusion^17^,^19^. The illusion of having an invisible hand is elicited through correlated tactile stimulation of a participant’s hidden hand and visual stimulation of a portion of empty space in front of the subject. Although the multisensory congruence rules and neural substrates in premotor-intraparietal-cerebellar cortices in the invisible hand illusion were characterized in detail in a previous study^16^, the intriguing question of whether the illusion can be extended beyond a single limb remained unanswered. The results of the study presented here suggest that the multisensory integrative mechanisms that underlie the invisible hand illusion can be generalized to an entire invisible body. This notion is compatible with the receptive field properties of visual-tactile-proprioceptive neurons found in the multisensory premotor and intraparietal cortices^30^: areas that are thought to be involved in generating sensations of body ownership^31^,^32^,^33^. These neurons feature visuo-tactile receptive fields anchored to the surface of one or multiple body parts (e.g., the arm, hand, face, trunk or combinations thereof)^30^,^34^,^35^,^36^ and sometimes even the whole body^37^ and its surrounding visual space. A subpopulation of these neurons exhibit object permanence, meaning that they encode the presence of a visual stimulus close to the body even though it is no longer visible^38^. It has been suggested that object permanence is important in regard to maintaining an accurate sense of the location of objects with respect to the body even in the absence of direct visual input or in the dark^38^,^39^. We speculate that these neuronal properties, which probably evolved from the ecological need to have a precise sense of the body in the absence of vision, allow a “body-shaped” portion of empty space to be attributed to the self in the present experiment. A possible neural mechanism is that premotor-intraparietal areas integrate visuo-tactile stimuli across body segments and contribute to the construction of the unified percept of owning an entire invisible body.

The elicitation of the invisible body illusion is, in many respects, similar to the mannequin (“body-swap”) illusion^17^, with the fundamental difference being the absence of visual input from a physical body (see Figure 1 for comparison). Experiments 2a and 2b showed that the invisible body and mannequin illusions are correlated with respect to subjective vividness and magnitude of threat-evoked SCRs. Based on these results, it seems likely that the invisible body and the mannequin illusions rely on similar visuo-tactile integration processes. Furthermore, we speculate that the multisensory areas that have been associated with experiencing the mannequin illusion; namely, the ventral premotor cortex, intraparietal sulcus, and the putamen^19^, are also involved in generating the experience of an invisible body. Nevertheless, there still exist important differences between the invisible body illusion and the mannequin illusion. First, we showed that the invisible body illusion has a unique effect on perceived body image (Experiment 3). In contrast to the mannequin illusion, the invisible body illusion affects the perceived physical nature of one’s body and induces a shift toward a hollow, transparent body image. This effect could not be explained by the mere visual input of an empty visual space, because synchronous visuo-tactile stimulation induced a significantly greater shift than asynchronous stimulation did. Interestingly, the mannequin illusion induced a significant body image shift in the opposite direction (i.e., toward a solid body image), speaking against the possibility that experiencing full-body illusions generally leads to higher ratings on this body image task. Second, we speculate that the mannequin and invisible body illusions differ in terms of their degree of generalizability across body segments. In all of the experiments in the present study, we stimulated five parts of the participants’ body (the arms, legs, and abdomen) because pilot anecdotal reports led us to believe that this mode of stimulation maximized the feeling of invisibility. However, it remains unclear whether the stimulation of one single invisible body part would lead to the experience of owning an entire invisible body, which has been shown to be true for the mannequin illusion^19^. Based on our anecdotal evidence and the observation that multisensory premotor-intraparietal areas are activated by the mere vision of a hand^40^,^41^, we speculate that the visual input of an intact physical body facilitates the spread of ownership across body segments and that the degree of generalizability is therefore higher for the mannequin illusion than for the invisible body illusion. This mechanism could possibly explain the observed decrease in SCR when the invisible body was threatened in Experiment 2b as compared to when the mannequin was threatened (although the interaction was body type × visuo-tactile temporal congruence was non-significant). These predictions should be tested in future studies.

Finally, we demonstrated that the invisible body illusion has unique effects on social cognition. It is reasonable to assume that this domain of cognitive functions evolved in relation to one’s body being represented as a physical entity that is visible to outside observers^42^,^43^. Being gazed upon constitutes a salient and important social cue^44^, and experiencing ownership of an invisible body could thus affect the socio-affective processing of such cues. We focused on the situation of standing in front of an audience. This situation is generally recognized as a stressful social event and is associated with an abnormally increased heart rate and level of anxiety in patients suffering from social phobia or performance anxiety^25^,^26^. We showed that the illusion of owning an invisible body, as compared to a mannequin’s body, is associated with decreased heart rate and level of subjective stress in response to standing in front of a group of strangers. This anxiety-reducing effect was absent in the asynchronous control condition and can therefore not be explained by effects that were non-specific to the illusion, such as the differences in visual input during the period preceding the “stressful social event.” These results support the dynamic interaction between neural representations of the bodily self and social brain areas involved in anxiety processing. Neuroimaging studies have consistently associated social anxiety responses with hyperactivity in the insula and amygdala, which might reflect an overactivation of the brain’s physiological fear system^45^,^46^. We hypothesize that the observed illusion-induced reduction of social anxiety is reflected in the neural interplay between multisensory representations of one’s own body in premotor-intraparietal areas^16^,^19^ and the fear-processing circuit involving the insula and amygdala^46^. Thus, the current study supports the hypothesis that own-body representations influence socio-cognitive processing^43^ by contributing to the emerging body of research on embodied cognition that suggests the existence of a causal link between central body representations and various higher cognitive functions. For instance, it has been demonstrated that illusory ownership of a dark-skinned rubber hand reduces implicit racial bias^47^, that efficient episodic-memory encoding requires perception of the world from the perspective of one’s own body^48^, and that illusory embodiment of a virtual child’s body causes implicit attitude changes^49^.

From an applied neuroscience perspective, our findings suggest that the invisible body illusion may play a role in the treatment of social anxiety. Cognitive-behavioral therapy is the dominating non-pharmacological treatment of this disorder^50^. Studies over the last decade have demonstrated that exposing patients to social situations within virtual environments can elicit social anxiety responses similar to those induced in real-world situations^16^,^20^,^21^, and that virtual reality exposure therapy (VRET) has a significant clinical effect^51^. In VRET, the patient is exposed to gradually increasing anxiety-provoking situations within a VR environment, with the aim being to habituate the anxiety response to the real-world stress-events. We propose that the illusion could be used as an initial step of VRET, in which the patient experiences a stressful situation under the perception that his or her body is invisible. Thereafter, the body solidness is gradually increased. We speculate that this strategy could have a good clinical effect in subgroups of patients in which the social anxiety response is contingent on negative emotions concerning one’s own body.

The present results could also have implications for neurological research on spinal phantom body experiences. To the best of our knowledge, the only perceptual experiences that share the key phenomenological characteristics of the present invisible body illusion are whole-body phantoms, which are, in some rare cases, reported by patients with cervical spinal cord injuries. These patients typically describe the experience of a phantom body, involving the trunk and all four extremities, that is spatially misaligned with respect to their paralyzed real body^52^,^53^,^54^. Although the interventions that give rise to the experience of a non-physical full-body differ between the perceptual illusion under investigation and full-body phantoms (multisensory stimulation vs. deafferation), investigating the cortical mechanisms underlying the invisible body illusion might provide clues as to why phantom bodies arise in the brain. It should be noted the experience of having an invisible body, such as whole-body phantoms, is distinct from the experience of *not having a body*, as in some cases of asomatognosia^55^. Patients suffering from asomatognosia often report that individual limbs, or even one half of body, has “faded away from consciousness”^56^,^57^. The participants in our study clearly denied the statement *“I could no longer feel my body”* while simultaneously agreeing to the statement “*It felt as if I had an invisible body”*, which strongly suggest that the invisible body illusion involves the feeling of having a body (although a completely transparent one) rather than the sensed body fading away from consciousness.

In conclusion, we have described a full-body illusion in which ownership of an invisible body was induced in healthy participants through the manipulation of the visual first-person perspective in conjunction with correlated visuo-tactile stimulation. Moreover, we have characterized certain perceptual and socio-cognitive consequences of this experience of invisibility, which have bearings on contemporary theories of body perception and embodied cognition. Finally, our findings revitalize the classic question raised by Plato almost two millennia ago^1^ regarding how the human mind would handle “the power” of invisibility from a social-moral perspective. This issue is becoming increasingly relevant today because of the emerging prospect of invisibility cloaking of an entire human body being made possible by modern materials science.

## Author Contributions

AG and HE developed the study concept. All authors contributed to the study design. Testing and data collection were performed by AG and ZA. AG and ZA performed the data analysis. AG drafted the manuscript, and ZA and HE provided critical revisions. All authors approved the final version of the manuscript for submission.

## Figures and Tables

**Table 1:** Questionnaire statements.

During the experiment …	
S1	I felt the touch of the brush in the empty space / on the mannequin* in the location where I saw the brush moving.
S2	It felt as if I had an invisible body / the mannequin’s body were my body*.
S3	I experienced that the touch I felt was caused by the brush moving in the empty space / touching the mannequin*.
S4	When I saw the brush moving, I experienced the touch on my back.
S5	It felt as if I had two bodies.
S6	I could no longer feel my body.

*This version of the questionnaire statements was used for the mannequin conditions in Experiment 2a.
